# Sustained-release losartan from peptide nanofibers promotes chondrogenesis

**DOI:** 10.3389/fbioe.2023.1122456

**Published:** 2023-02-06

**Authors:** Kohei Yamaura, Nicholas A. Sather, Anna Metlushko, Haruki Nishimura, Radoslav Z. Pavlović, Sealy Hambright, Sudheer K. Ravuri, Marc J. Philippon, Samuel I. Stupp, Chelsea S. Bahney, Johnny Huard

**Affiliations:** ^1^ Center for Regenerative and Personalized Medicine, Steadman Philippon Research Institute, Vail, CO, United States; ^2^ Simpson Querrey Institute for Bionanotechnology, Northwestern University, Chicago, IL, United States; ^3^ The Steadman Clinic, Vail, CO, United States; ^4^ The Orthopaedic Trauma Institute, University of California, San Francisco (UCSF), San Francisco, CA, United States

**Keywords:** osteoarthritis, chondrocytes, losartan, peptide amphiphile nanofibers, sustained release, TGF-β

## Abstract

**Introduction:** The central pathologic feature of osteoarthritis (OA) is the progressive loss of articular cartilage, which has a limited regenerative capacity. The TGF-β1 inhibitor, losartan, can improve cartilage repair by promoting hyaline rather that fibrous cartilage tissue regeneration. However, there are concerns about side effects associated with oral administration and short retention within the joint following intra-articular injections. To facilitate local and sustained intra-articular losartan delivery we have designed an injectable peptide amphiphile (PA) nanofiber that binds losartan. The aims of this study are to characterize the release kinetics of losartan from two different PA nanofiber compositions followed by testing pro-regenerative bioactivity on chondrocytes.

**Methods:** We tested the impact of electrostatic interactions on nanostructure morphology and release kinetics of the negatively charged losartan molecule from either a positively or negatively charged PA nanofiber. Subsequently, cytotoxicity and bioactivity were evaluated *in vitro* in both normal and an IL-1β-induced OA chondrocyte model using ATDC5.

**Results:** Both nanofiber systems promoted cell proliferation but that the positively-charged nanofibers also significantly increased glycosaminoglycans production. Furthermore, gene expression analysis suggested that losartan-encapsulated nanofibers had significant anti-inflammatory, anti-degenerative, and cartilage regenerative effects by significantly blocking TGF-β1 in this *in vitro* system.

**Discussion:** The results of this study demonstrated that positively charged losartan sustained-release nanofibers may be a novel and useful treatment for cartilage regeneration and OA by blocking TGF-β1.

## Introduction

Articular cartilage is the connective tissue that covers the epiphyseal surface of articular bones. Articular cartilage has specialized viscoelastic properties to facilitate load transfer across the subchondral bone and support smooth, lubricated articulation within the joint. Articular cartilage consists primarily of chondrocytes and an extracellular matrix composed of type II collagen, proteoglycans, and glycosaminoglycans (GAGs), (Cheng et al., 2013), (Armiento et al., 2018). Cartilage tissue has low mitotic activity (Lefebvre and Smits, 2005) and limited self-repair capacity (van Osch et al., 2009) due to its non-vascular nature. Osteoarthritis (OA) is a disease in which there is an irreversible loss of articular cartilage that affects approximately 240 million people worldwide (Nelson, 2018) and significantly disrupts the patient’s activities of daily living and quality of life (Aletaha et al., 2011), (Linn et al., 2011). Currently, mild to moderate OA is treated with conservative therapies such as anti-inflammatory drugs and hyaluronic acid injections in the early stages. Surgical arthroplasty is typically required to restore patient mobility in the later stages of disease as an effective treatment for cartilage regeneration has not yet been established.

While transforming growth factor β1 (TGF-β1) is known to be essential for articular cartilage homeostasis (Blaney Davidson et al., 2007), (Shen et al., 2013), TGF-β1 has also been reported to be involved in OA. OA chondrocytes overproduce TGF-β1 (van de Laar et al., 2011), and intra-articular injections of TGF-β1 have been shown to induce OA (Bakker et al., 2001), (Itayem et al., 1999). Recently, losartan, an angiotensin II receptor antagonist for hypertension, has attracted attention for its ability to inhibit fibrocartilage formation and promote articular cartilage repair by suppressing TGF-β1 (Utsunomiya et al., 2020). However, there is concern about potential side effects of oral administration of the drug, which is an antihypertensive agent. While Logan *et al* showed that intra-articular injection of losartan promotes cartilage regeneration, they also reported that higher doses of losartan inhibit cartilage regeneration (Logan et al., 2021), suggesting that there is a dose-dependent regenerative response of cartilage to losartan. Furthermore, as a low molecular weight (Mw) drug (Mw < 10,000 Da), losartan can easily diffuse through the interstitium and capillary walls (Gerwin et al., 2006) and is expected to disappear from the joint in a few days. Previous reports investigating the half-life of local anesthetics, antipyretic analgesics, and antibacterial agents in joints have shown that, depending on the type, it ranged from approximately 1.1–6.25 h (Owen et al., 1994), (Lescun et al., 2000). To optimize losartan administration, addressing both the drugs’ short half-life, and localization of the drug will improve regenerative function. One approach to generating local and sustained release of losartan within the intra-articular space is to attach the drug to a biomaterial scaffold that can be injected into the intra-articular space.

Peptide amphiphile (PA) molecules are a broad group of molecules that typically feature covalently linked hydrophilic peptide segments and hydrophobic segments such as alkyl chains (Behanna et al., 2005), (Claussen et al., 2003). These amphiphilic molecules are designed to self-assemble from aqueous solution into high aspect ratio supramolecular nanofibers that mimic the structure and function of natural extracellular matrices The nanofibers form a stable dispersion in water but can be further crosslinked into self-supporting gels composed of an interconnected network of nanofibers (Hartgerink et al., 2002), (Hendricks et al., 2017). A previous study showed that co-assembly of PA molecules exhibiting epitopes that bind to TGF-β1 promotes cartilage regeneration in an articular cartilage defect rabbit model (Shah et al., 2010). Local administration of drugs using self-assembling peptide nanofiber gels has been widely investigated as an alternative to reduce systemic side effects by increasing bioavailability at the target organ, especially in anticancer drugs (Kim et al., 2009), (Cinar et al., 2016). Cinar et al. (2016) reported that encapsulation of doxorubicin, a chemotherapeutic agent widely used in the treatment of breast cancer, in PA nanofiber gels suppressed tumor growth rates compared to direct injection.

In this study, we modified the peptide composition of our previously published PA nanofibers (Shah et al., 2010), (Lewis et al., 2020) to achieve an injectable nanofiber formulation that can bind and slowly release losartan. We hypothesize that the PA nanofiber chemistry can be modified to achieve sustained release of losartan over a two-week period and promote chondrocyte proliferation and chondrogenesis *in vitro*.

## Material and methods

### Preparation of losartan-encapsulated PA nanofibers

**FIGURE 1:**
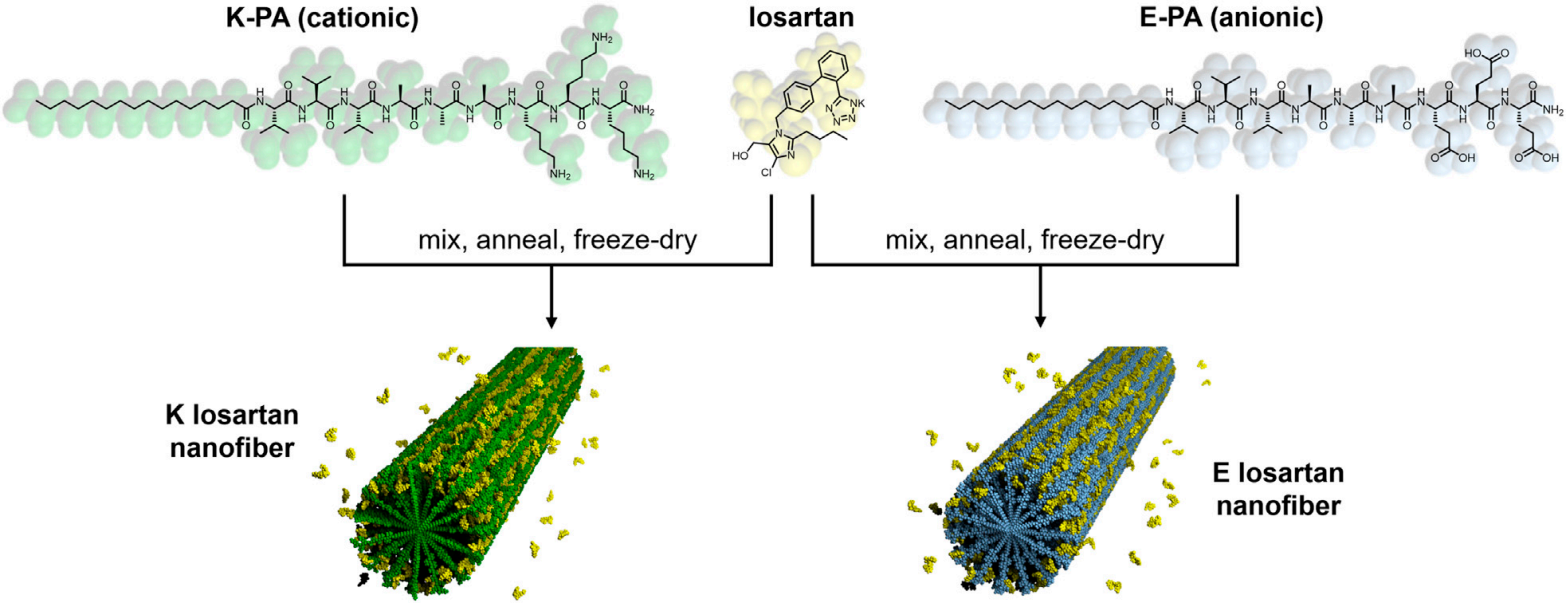
Chemical structure and schematic of losartan sustained release PA nanofibers. Positively charged K-PA (green) and negatively charged E-PA (light blue) are co-assembled in water with losartan (yellow) to form K losartan nanofibers and E losartan nanofibers, respectively.

A total of eight nanofiber formulations **(** Figure 1) were prepared by combining four different losartan potassium (Sigma-Aldrich, St. Louis, MO, United States) doses (0, 0.5, 5, and 50 mg/mL) and two PA molecules with opposite charge. Cationic PA molecules (K-PA) with the sequence palmitoyl-VVVAAAKKK and anionic PA molecules (E-PA) with the sequence palmitoyl-VVVAAAEEE were synthesized by solid phase peptide synthesis as described previously (Sather et al., 2021), and had a purity of more than 95% by liquid chromatography-mass spectroscopy (LC-MS). PA nanofiber assemblies at each losartan dose were prepared by first solubilizing K-PA or E-PA at 10 mg/mL in 150 mM NaCl and 3 mM KCl and pH adjusting to 7.4 using 1 M NaOH before adding various amounts of losartan powder. The solutions were vortex mixed, sonicated for 30 min, and then thermally annealed in a water bath at 80°C for 30 min followed by slow cooling to room temperature overnight. The annealed solutions were then frozen in liquid nitrogen and freeze-dried for 24 h, with the resulting lyophilized powders stored at 4°C until further use.

### Physical and chemical characterization of losartan sustained release nanofibers

Samples for transmission electron microscopy (TEM) were resuspended at a PA concentration of 10 mg/mL and then diluted 10-fold–1 mg/mL immediately before 10 µL of sample solution was transferred to plasma-cleaned 300-mesh copper grids with lacey carbon support (Electron Microscopy Science). Samples were stained with 2% uranyl acetate. Imaging was performed using a FEI Spirit G2 TEM working at 120 kV accelerating voltage.

Nuclear magnetic resonance (NMR) spectroscopy was performed on a Bruker Neo 600 MHz system with QCI-F cryoprobe at 298 K. The lyophilized powders from the sample preparation protocol above were resuspended in D_2_O at 10 mg/mL PA concentration and 5 mg/mL losartan concentration. This corresponds to 8.7 mM E-PA, 8.7 mM K-PA, and 10.8 mM losartan.

### Drug release studies of losartan from nanofibers

Absorbance spectroscopy was used to measure losartan release from PA nanofibers. Freeze dried PA-losartan powders were reconstituted in milli-Q H_2_O at 10 mg/mL PA and 5 mg/mL losartan and divided into 100 μL aliquots in 1 mL Eppendorf tubes. PA nanofiber hydrogels were formed by adding 50 μL of either 1x phosphate buffered saline (PBS) to K-PA solutions or 25 mM CaCl_2_ in 1x tris buffered saline (TBS) to E-PA solutions. Samples were incubated at 37°C for 1 h to allow for gel formation at the bottom of the tube. Then 400 μL of release buffer (1x PBS for K-PA gels and 10 mM CaCl_2_ in 1x TBS for E-PA gels) was added to each tube and the samples were incubated at 37°C. Every 24 h, 400 μL of release buffer was removed from each tube for absorbance spectroscopy measurements and replaced with fresh release buffer. All release experiments were repeated in triplicate. Absorbance spectra were collected using an Ocean Optics USB4000-UV-VIS spectrometer, with data update rate = 100 μs, scans to average = 100, and boxcar width = 2. Release buffer samples at each time point were loaded into a quartz cuvette with 1 cm pathlength, and fresh release buffer solutions were used for background subtraction. The primary losartan peak absorbance at 239 nm was measured and compared to a calibration curve of known concentrations of losartan to determine the amount of losartan released at each time point. The absorbance in the release buffer at the t = 0 time point was used to calculate the drug loading efficiency.

### Cell culture

**FIGURE 2:**
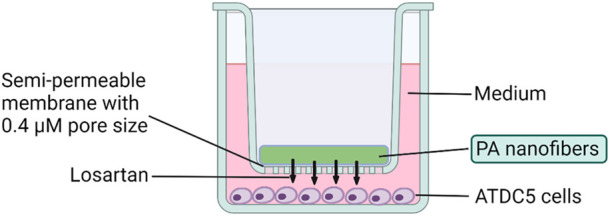
Cell culture method using losartan sustained release nanofibers. Transwell plates were used for the culture of ATDC5 cells. Chondrocytes were cultured at the bottom of the plate, and each nanofiber was implanted on a 0.4 μm semipermeable membrane.

The ATDC5 cell line is a continuous long-term culture line derived from mouse teratocarcinoma that is commonly used as a model for *in vitro* chondrocyte research (Yao and Wang, 2013). ATDC5 (Sigma-Aldrich, St. Louis, MO, United States) cells were plated at a density of 1 × 10^4^ cells/well on the bottom of a 24-well transwell plate (Corning, Glendale, AZ, United States) (Figure 2). These cells were cultured for 48 h in a “complete chondrogenic media” defined as Dulbecco’s Modified Eagle Medium (DMEM)/Nutrient F-12 Ham (Thermo Fisher, Winsor, NJ, United States), containing 5% fetal bovine serum (FBS) (Thermo Fisher, Winsor, NJ, United States), and 1% penicillin and streptomycin (Invitrogen, Waltham, MA, United States), followed by 1% insulin-transferrin-sodium selenite (ITS) (Sigma-Aldrich, St. Louis, MO, United States) to support chondrogenic differentiation as previously published (Chen et al., 2020). Total of 12 groups were divided by losartan dose (0, 0.5, 5, and 50 mg/mL) and presence/absence of positively charged K or negatively charged E nanofibers (Table 1). Each nanofiber was implanted on a 0.4 μm semipermeable membrane (Corning, Glendale, AZ, United States) (Figure 2). Losartan solution without nanofibers was prepared by dissolving powdered losartan, identical to the drug contained in the nanofibers, in culture medium. The culture was continued for 2 weeks at 5% CO_2_, 21% O_2_, and 37°C, with the nanofibers remaining untouched and the medium changed every 3 days from the start of nanofiber treatment.

**TABLE 1:** Grouped by combination of losartan and nanofibers.

Group	Control	K0	K1	K2	K3	E0	E1	E2	E3	L1	L2	L3
Nanofibers	–	K	K	K	K	E	E	E	E	–	–	–
Losartan (mg/mL)	0	0	0.5	5.0	50	0	0.5	5.0	50	0.5	5.0	50

K nanofibers: Positively charged nanofibers.

E nanofibers: Negatively charged nanofibers.

### Cell proliferation and toxicity assay

Cell proliferation of ATDC5 cells in each group was evaluated by PrestoBlue assay (Invitrogen, Waltham, MA, United States) according to the manufacturer’s protocol (Boncler et al., 2014). This non-destructive assay can approximate the number of living cells present based on the mitochondrial reduction of resazurin dye (blue, non-fluorescent) to resorufin (red, highly fluorescent). Cell proliferation was semi-quantitated as a longitudinal fold change from control group at 24 h, 5 days, and 14 days after treatment follow a 1 h of reaction with PrestoBlue at 37°C. Fluorescence in each well is quantified by measuring absorbance at 570 nm using a microplate reader (Infinite 200 Pro, Tecan, Männedorf, Switzerland) and normalizing by the value at 600 nm (*n* = 8 per group).

### *In vitro* osteoarthritis induction

Twenty four hours after plating ATDC cells (1 × 10^4^ cells/well) into a 24-well transwell plate, 10 ng/mL interleukin (IL)-1β (Peprotech, Cranbury, NJ, United States) was added to the complete chondrogenic media for 24 h to model OA *in vitro* as previously published (Deng et al., 2021). The cells were then washed twice with phosphate buffered saline (PBS) (Thermo Fisher, Winsor, NJ, United States) and cultured again for 24 h in complete chondrogenic media with or without losartan delivery.

### Extracellular matrix (ECM) production

GAG production was quantitatively evaluated by measuring the reaction of GAG with 1,9-dimethyl-methylene blue (DMMB) (Sigma-Aldrich, St. Louis, MO, United States) 14 days after initiation of treatment according to the well-defined method. Briefly, each well of a 96-well plate was filled with 15 μL of supernatant, and 150 μL of DMMB solution. GAGs production was measured at 575 mm with a microplate reader (Infinite 200 Pro, Tecan, Männedorf, Switzerland) using a standard curve obtained with shark chondroitin sulfate (Sigma-Aldrich, St. Louis, MO, United States). GAGs production was standardized by calculating GAGs production per cell unit using the number of cells estimated by PrestoBlue assay for each sample (*n* = 8 per group).

### Gene expression analysis

Total RNA was isolated from ATDC5 cells using TRizol Reagent (Thermo Fisher, Winsor, NJ, United States) and reverse transcribed according to the manufacturer’s protocol using iScript reverse transcription supermix cDNA synthesis kit (Bio-Rad, Hercules, CA, United States) 14 days after treatment. qRT-PCR was performed using the Applied Biosystems™ SYBR™ Green Assay Kit (Thermo Fisher, Winsor, NJ, United States) and Applied Biosystems StepOnePlus RT-PCR thermocycler (Applied Biosystems, San Francisco, CA, United States). Francisco, CA, United States). Primers were designed using PRIMER-Blast (NCBI) and the sequences are listed in Table 2. The evaluated mRNAs were the inflammation marker interleukin-6 (*Il6*), the hypertrophic chondrocyte marker type X collagen (*colXa1*), matrix metalloproteinase-13 (*mmp13*), and the chondrocyte markers type II collagen (*colIIa1*) and Aggrecan (*acan*). To evaluate the inhibitory effect of losartan on TGF-β1, mRNA expression of *tgfβ1* was also evaluated. These genes of interest were normalized to the expression of the housekeeping gene glyceraldehyde 3-phosphate dehydrogenase (*gapdh*) expression. Relative gene expression was calculated compared to the control culture condition (no nanofibers or losartan) using the 2^−ΔΔCT^ method (*n* = 8 per group).

**TABLE 2:** Primer sequences for qRT-PCR.

Gene	Oligonucleotide sequence
*Il6*	Forward 5’TGA​TGG​ATG​CTA​CCA​AAC​TGG​A 3’
	Reverse 5’TGT​GAC​TCC​AGC​TTA​TCT​CTT​GG 3’
*colIIa1*	Forward 5’GCC​AGG​ATG​CCC​GAA​AAT​TAG 3’
	Reverse 5’ACG​ATC​ACC​TCT​GGG​TCC​TT 3’
*colXa1*	Forward 5’TTG​CTA​GCC​CCA​AGA​CAC​AA 3’
	Reverse 5’GTC​CAG​GAC​TTC​CAT​AGC​CT 3’
*acan*	Forward 5’TTG​CAG​ACA​TTG​ACG​AGT​GC 3’
	Reverse 5’TTA​GTC​CAC​CCC​TCC​TCA​CA 3’
*mmp13*	Forward 5’ACC​CAG​CCC​TAT​CCC​TTG​AT 3’
	Reverse 5’GGT​CAC​GGG​ATG​GAT​GTT​CA 3’
*tgfβ1*	Forward 5’ACT​GGA​GTT​GTA​CGG​CAG​TG 3’
	Reverse 5’GGG​GCT​GAT​CCC​GTT​GAT​TT 3’
*gapdh*	Forward5’ CCC​TTA​AGA​GGG​ATG​CTG​CC 3’
	Reverse 5’TAC​GGC​CAA​ATC​CGT​TCA​CA 3′

### Statistical analyses

All experiments were repeated at least twice with a *n* = 3 replicates per experiment. All plots and statistical comparisons were made using GraphPad Prism version 9.4.0 (GraphPad Software, La Jolla, CA, United States). Bar plots represent the mean ± standard deviation with each individual replicate plotted as a dot on the bar graph. The groups were compared by analysis of variance (ANOVA) with statistical significance set at *p* < 0.05. For significantly different groups, Tukey’s Honestly Significant Different (HSD) multiple comparison *post hoc* testing was performed with statistical significance again set at *p* < 0.05.

## Results

### Positively charged PA nanofibers produced a sustained release of losartan

**FIGURE 3:**
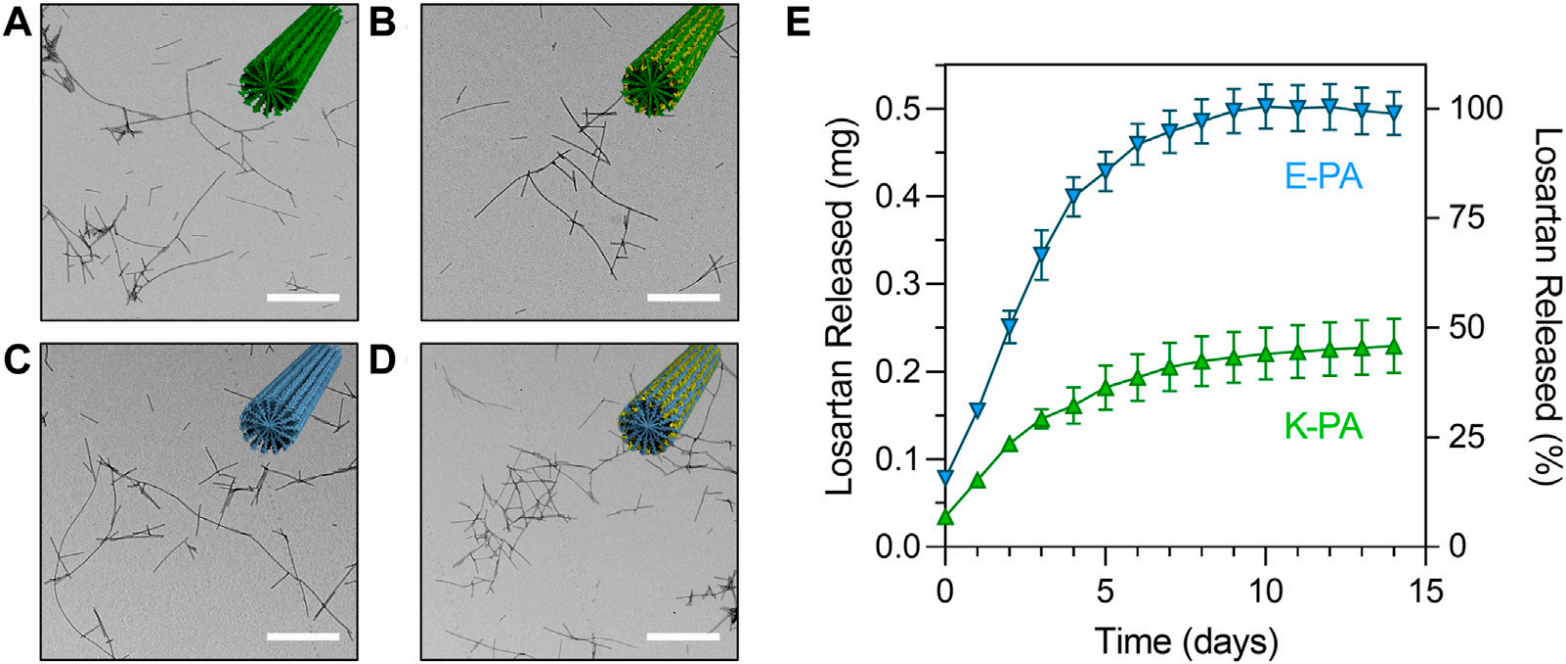
Structure and release profile of losartan sustained release nanofibers. Transmission electron micrographs of **(A)** K-PA fibers, **(B)** K-PA fibers with 5 mg/mL losartan, **(C)** E-PA fibers, and **(D)** E-PA fibers with 5 mg/mL losartan. Scale bars = 500 nm. Insets show illustrations of nanofiber assemblies with K-PA in green, E-PA in light blue, and losartan in yellow. **(E)** Losartan release profile from K-PA and E-PA fibers for a losartan concentration of 5 mg/mL, which corresponds to 0.5 mg of total losartan encapsulated in each experiment.

Losartan encapsulated PA nanofibers were prepared by physical mixing of PA and losartan solutions followed by thermal annealing to drive co-assembly of losartan into the PA supramolecular structure (see Figure 1). We hypothesized that hydrophobic interactions between losartan and the aliphatic core of the nanofiber would initiate encapsulation and that opposing electrostatic effects on positively charged K-PA vs. negatively charged E-PA nanofibers may alter the binding affinity to the drug. In TEM imaging, we observed similar fiber length distributions (∼200–2,000 nm) for K-PA and E-PA nanofibers both with and without losartan (Figures 3A–D). We note that these nanofiber lengths are shorter than previously reported PA nanofibers from our group because the lyophilization step after thermal annealing can fracture the supramolecular fibers (Chen et al., 2018). However, for our target therapy of a liquid injection in the intra-articular space, we preferred the shorter nanostructures to minimize aggregation or gelation.

We first measured the drug loading efficiency and found that both K-PA and E-PA nanofibers were highly effective at encapsulating losartan (93% loading for K-PA and 84% loading for E-PA). Then, we measured the release of losartan from positively charged K-PA and negatively charged E-PA nanofibers by monitoring the losartan absorbance in a release buffer over the course of two weeks. We found that both nanofibers offered a sustained release profile but that negatively charged E-PA nanofibers released losartan at approximately twice as fast as the positively charged K-PA nanofibers (Figure 3E). At a moderate dose of losartan (5 mg/mL), all of the losartan was released from negatively charged E-PA after 1 week, while positively charged K-PA nanofibers still retained ∼55% of the losartan after 2 weeks.

**FIGURE 4:**
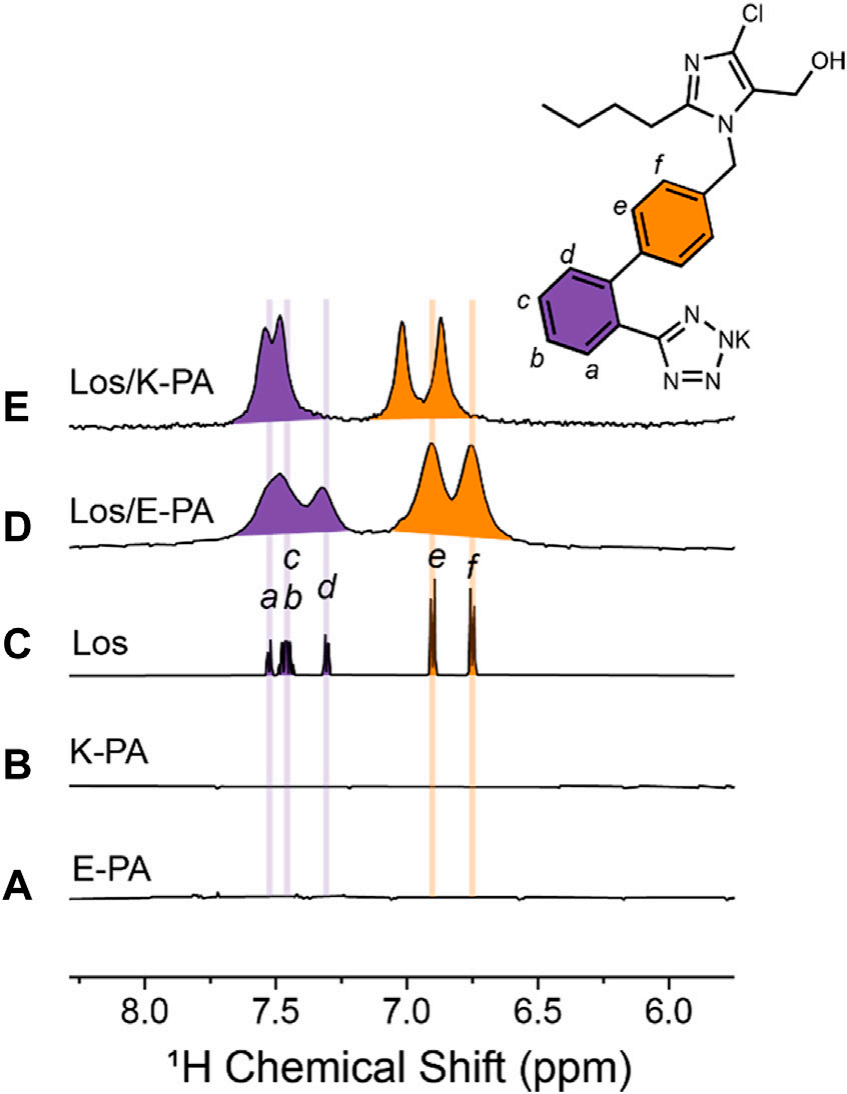
Losartan-PA binding interactions probed by NMR spectroscopy. Region of ^1^H NMR spectra measured for **(A)** 10 mg/mL E-PA, **(B)** 10 mg/mL K-PA, **(C)** 5 mg/mL losartan, **(D)** 5 mg/mL losartan, and 10 mg/mL E-PA, and **(E)** 5 mg/mL losartan, and 10 mg/mL K-PA. Note that signals corresponding to aromatic protons are colored purple and orange for ortho- and para-disubstituted benzene rings, respectively.

We utilized ^1^H NMR spectroscopy in solution to probe the binding of losartan to E− and K-PA nanofibers. Our analysis focused on the chemical shift region featuring the aromatic protons from losartan (Figure 4), in which E− and K-PA assemblies alone show no signal (Figures 4A, B). The spectrum of free losartan shows a set of sharp resonances that correspond to *ortho*- (purple) and *para-*disubstituted (orange) benzene moieties (Figure 4C). This is expected since losartan, a small molecule in the monomeric state, can undergo fast rotational tumbling in solution. In contrast, the ^1^H NMR signals from losartan measured in the samples of the drug-PA co-assemblies are significantly broadened out. This is due to chemical exchange of the drug molecules among the *free* and PA nanofiber *bound* states (losartan_*free*_ ⇌ losartan_*bound*_), indicating encapsulation of losartan into the PA nanofiber assembly. It is expected that losartan’s bound state shows faster transverse relaxation rate (*R*_*2*_) in comparison to its free state (*R*_*2,free*_ < *R*_*2,bound*_), considering the larger size of the losartan-nanofiber assembly compared to monomeric losartan, which causes the excessive NMR line broadening of the losartan signals in the samples where *P*As are present. Importantly, the positively charged K-PA/losartan spectrum shows signals shifted downfield relative to those measured for losartan alone and in the negatively charged E-PA/losartan co-assembly. This shift can be attributed to attractive electrostatic interactions between the tetrazolate segment of losartan and the lysine ammonium moieties (NH_3_^+^···tetrazolate), which are present in positively charged K-PA, but not in negatively charged E-PA. Thus, in addition to the hydrophobic effect that drives the PA nanofiber/losartan association, unique charge complementarity present within the K-PA/losartan system enhances the persistency of K-PA/losartan relative to the E-PA/losartan co-assembly, which is in line with the losartan release experiments where K-PA releases losartan slower than E-PA.

### Losartan non-ofibers are non-cytotoxic and promote cell proliferation in a losartan-independent fashion

**FIGURE 5:**
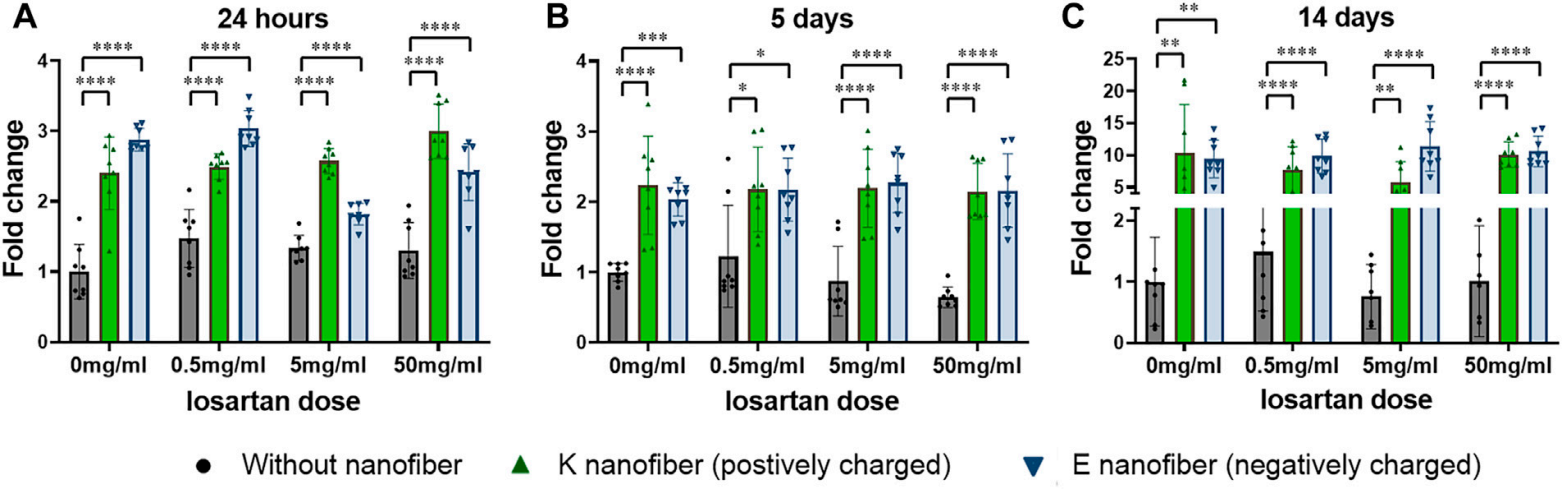
Nanofibers promote cell proliferation in a losartan-independent fashion. Cell proliferation was significantly increased in both the positively charged K and negatively charged E nanofibers at all losartan doses after **(A)** 24 h, **(B)** 5 days, or **(C)** 14 days of treatment. ●(black); without nanofiber, ▲(green); K nanofiber (positively charged), and ▼(blue); E nanofibers (negatively charged). *N* = 8 per each group.

Cytotoxicity and ATDC5 cell proliferation was evaluated at 24 h, 5 days, and 14 days in groups treated according to the schema shown in Table 1 and compared to a control group that was not treated with PA nanofibers or losartan (Figure 5). No cytotoxicity was observed in any of the treatment groups. We found that both positively and negatively charged PA nanofibers significantly increased cell numbers at all time points compared to no nanofibers at each losartan dose. The addition of losartan did not result in a significant difference in cell proliferation at any dose.

### Positively charged nanofibers promote chondrogenesis

**FIGURE 6:**
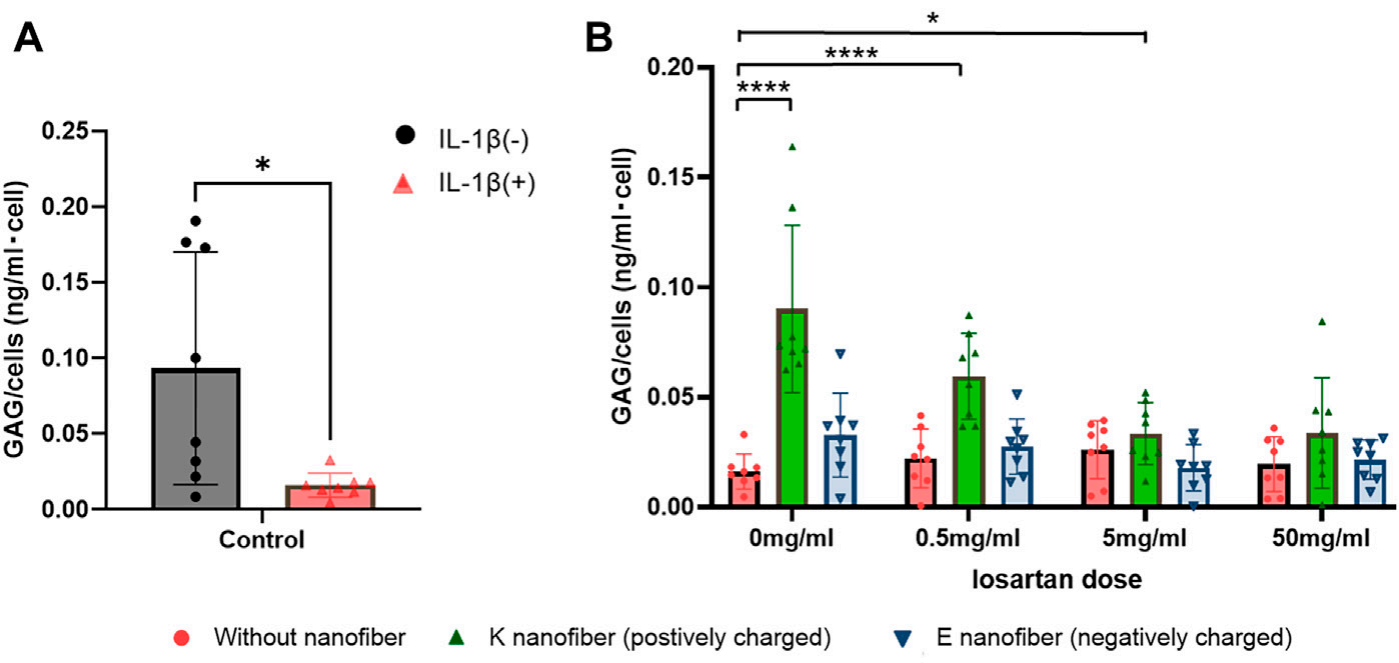
Positively charged nanofibers with low doses of losartan promote GAG production. **(A)** GAG production is signicantly decreased in OA-induced chondrocytes 24 h after treatment with IL-1β. ●(black); without IL-1β and ▲(red); with IL-1β. *; *p* < 0.05. *N* = 8 per each group. **(B)** Comparison of GAG production per cell at each losartan dose in the OA-induced chondrocytes. ●(red); without nanofiber, ▲(green); K nanofiber (positively charged), and ▼(blue); E nanofibers (negatively charged). *; *p* < 0.05 and ****; *p* < 0.001. *N* = 8 per each group.

Bioactivity and the regenerative effect of the nanofibers with or without losartan was tested for our clinical application by using an established *in vitro* model of OA (Deng et al., 2021). We first confirmed that, in our hands, 24 h of IL-1β (10 ng/mL) leads to reduced GAG production (Figure 6A). Subsequently, GAG production in the OA-induced chondrocytes was evaluated following losartan treatment either with or without the PA nanofibers. Significant increase in GAG production was observed with the positively charged K-PA nanofibers alone or when dosed with low (0.5 mg/mL) or moderate (5 mg/mL) losartan compared to controls without losartan or nanofibers (Figure 6B, green bars). The negatively charged E-PA nanofibers had no significant impact on GAG production compared to controls without nanofibers and losartan (Figure 6B, blue bars).

**FIGURE 7:**
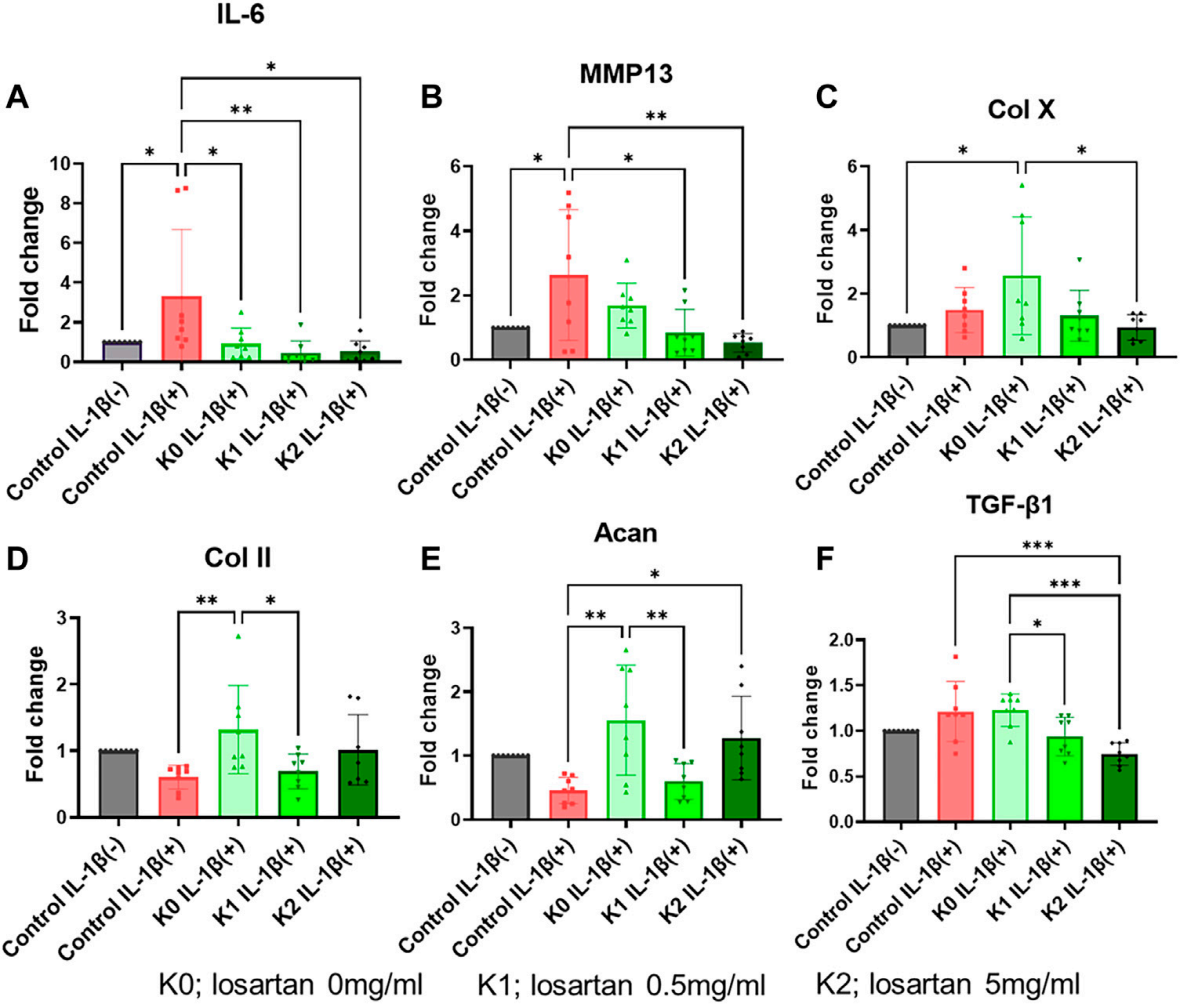
Positively charged nanofibers have pro-regenerative impact on OA-induced chondrocytes. Relative expression of **(A)** *interleukin-6*, **(B)** *mmp13*, **(C)** *collagen X*, **(D)** *collagen II*, and **(E)** *tgf-β1* in chondrocytes following treatment with IL-1β (red bar) or with varying doses of losartan encapsulated in positively charged K nanofibers (green bars) compared to untreated controls (grey bar). K0; losartan 0 mg/ml, K1; losartan 0.5 mg/dL, and K2; losartan 5 mg/dL *; *p* < 0.05, **; *p* < 0.01, and ***; *p* < 0.001. *N* = 8 per each group.

Chondrocyte response to the losartan nanofibers was also evaluated by looking at relative changes in expression of canonical hyaline cartilage markers (*col2a1*, *acan*), the pro-inflammatory marker *il6*, and the chondrocyte hypertrophy and degradation markers (*colXa1, mmp13*). Induction of an osteoarthritis-like phenotype in the ATDC5 chondrocytes was confirmed by a significant upregulation of *il6, mmp13*, and *colx10a1* (Figures 7A–C) and a decrease in *col2a1* and *acan* (Figures 7D, E) expression following treatment with IL-1β. Since experimentation up to this point supports a more beneficial effect of the positively charged K-PA nanofibers over the negatively charged E-PA nanofibers, gene expression analysis subsequently focused only on the positively charged K-PA nanofibers at the low and medium doses of losartan. The positively charged nanofibers themselves significantly suppressed *il6* expression, and this stayed low, but not significantly different with the addition of losartan (Figure 7A). Conversely, positively charged nanofibers did not significantly change *mmp13* expression, but increasing doses of losartan resulted in a dose dependent decrease in expression of both the metalloproteinase (*mmp13*) and hypertrophy (*col10a1*) marker (Figures 7B, C). A pro-regenerative response was stimulated by the addition of the positively charged nanofibers, as evident by the significantly increased expression of *col2a1* and *acan* (Figures 7D, E). Bioactivity of the losartan as a TGFβ antagonist was further confirmed by showing a dose-dependent decrease in *tgfβ1* expression with increasing losartan delivery (Figure 7F).

## Discussion

The results of this study showed that slower sustained release of losartan was achieved with our designed PA nanofibers, especially in the positively charged K-PA nanofibers. For this study, we chose to modify the previously used PA nanofiber chemistry to create an injectable, rather than surgically implanted, system that could bind, and slowly release losartan. We expected that PA nanofibers could readily encapsulate losartan *via* hydrophobic interactions between the biphenyl and butyl groups on losartan and the aliphatic core of the nanofiber. Release of losartan was effectively altered by using a different composition of hydrophilic terminal peptides in order to create nanofibers with either a net positive or net negative charge. Peptide composition and charge distribution did not influence the ability of the nanofibers to self-assemble with both compositions showing similar nanostructure by TEM. Importantly the positively charged nanofibers led to a significantly prolonged release of losartan with delivery sustained for approximately 2 weeks. This result suggests that in addition to hydrophobic encapsulation, positively charged nanofibers supported an electrostatic interaction with the negatively charged losartan (Al-Majed et al., 2015) that enhanced the binding affinity between the peptide amphiphile molecules and the drug. NMR studies confirmed the association of losartan with the PA nanofiber assemblies and revealed the additional electrostatic interaction in positively charged K-PA/losartan nanofibers that is responsible for the slower drug release compared to negatively charged E-PA/losartan nanofibers.

Losartan, an angiotensin II receptor antagonist, has previously been reported to have a beneficial effect on OA or cartilage defects by promoting hyaline cartilage tissue regeneration and decreasing fibrosis through inhibition of TGF-β1 (Utsunomiya et al., 2020), (Logan et al., 2021), (Deng et al., 2021), (Chen et al., 2015). Utsunomiya et al (Utsunomiya et al., 2020) reported that oral administration of losartan suppressed fibrocartilage and increased hyaline cartilage in a rabbit model of cartilage defect. However, oral administration of losartan also potentially carries the risk of hypotension as a side effect of this drug, which is clinically used as an antihypertensive. Deng et al. (2021) compared oral administration and intra-articular delivery of losartan and found that intra-articular injection had a stronger regenerative effect. However, Logan et al. (2021) found that intra-articular losartan injections at high doses may have adverse effects on cartilage. Considering the adverse effects of this high dose of losartan, as well as the relatively early elimination of the drug from the joint (Gerwin et al., 2006), we utilized PA nanofibers as a drug delivery system for localized and sustained release of losartan within the intra-articular space. In a previous study, a PA nanofiber gel loaded with TGF-β1 was implanted within cartilage defects as a scaffold designed to support cartilage tissue formation (Shah et al., 2010). The results of that study showed that the PA nanofibers aided the survival of human mesenchymal stem cells and promoted chondrogenic differentiation *in vitro* and promoted articular cartilage regeneration in a rabbit chondral defect model *in vivo*, even without the addition of exogenous growth factors.

A number of previous *in vitro* studies on peptide amphiphile nanofiber scaffolds have demonstrated their ability to induce cell proliferation, differentiation, and extracellular matrix production (Kisiday et al., 2002), (Liu et al., 2010), (Rubert Pérez et al., 2017), (Ji et al., 2018), (Webber et al., 2011). Self-assembled peptide amphiphile nanofibers have been reported to maintain chondrocyte morphology and promote cell division by encapsulating chondrocytes (Kisiday et al., 2002). The results of this study also showed that PA nanofibers, of either charge, significantly increased chondrocyte proliferation. Interestingly, this effect was independent of losartan delivery and no negative cellular effect or cytotoxicity was observed through the addition of any of the losartan doses tested in this study (0.5–50 mg/mL). A previous report showed that low doses of losartan increased chondrocyte proliferation *in vitro*, while high doses of losartan had a detrimental effect of decreasing cell proliferation (Deng et al., 2021).

As for ECM production, the results of this study showed that positively charged K-PA nanofibers without losartan and with low (0.5 mg/mL) to moderate (5 mg/mL) losartan significantly increased GAG production for OA cells. In contrast, negatively charged E-PA nanofibers did not show a significant increase in GAG production regardless of losartan dose. This result may suggest that positively charged K-PA nanofibers may provide more effective ECM production ability due to their electrical complementarity with GAGs, considering the characteristics of negatively charged GAGs (DiDomenico and Bonassar, 2018). Positively charged K-PA nanofibers have been reported to take advantage of this negatively charged nature of intra-articular tissues to more efficiently treat intra-articular joints (Vedadghavami et al., 2019), (Bajpayee et al., 2017). Furthermore, even at the mRNA level, *acan* expression was significantly increased in positively charged K-PA nanofibers with 5 mg/mL losartan and without losartan, and *col2a1* expression was significantly increased in positively charged K-PA nanofibers without losartan, supporting the results of the GAG assay.

Deng et al. (2021) reported that losartan suppressed the expression of anti-inflammatory markers such as IL6 and antidegenerative markers such as MMP13 in an OA cell model. In this study, positively charged K-PA nanofibers with low (0.5 mg/mL) to moderate (5 mg/mL) doses of losartan also significantly suppressed *il6* and *mmp13* expression. Interestingly, positively charged K-PA nanofibers without losartan did not suppress the expression of *mmp13*, whereas it significantly increased the expression of *col10a1*, a marker of hypertrophic chondrocytes, which is associated with chondrocyte OA (He et al., 2019). The results suggested that low doses of losartan, but not PA nanofibers, had an effective inhibitory effect on hypertrophic differentiation and inflammation of chondrocytes in the OA cell model. Previous reports have described various mechanisms by which losartan has shown beneficial effects on cartilage, but most are mainly based on the inhibition of TGF-β1 (Utsunomiya et al., 2020), (Logan et al., 2021), (Deng et al., 2021), (Chen et al., 2015), (Thomas et al., 2019). Deng et al. (2021) reported that losartan inhibited TGF-β1 in OA chondrocytes. Similarly, our study showed *tgfβ1* was significantly inhibited by the positively charged K-PA nanofibers with low (0.5 mg/mL) to moderate (5 mg/mL) doses of losartan.

The limitation of the current study is that the losartan nanofibers are only validated *in vitro*. The authors acknowledge that further testing will be required to demonstrate *in vivo* efficacy. However, given prior studies with losartan are rigorously tested in a rabbit chondral defect model, this validation testing is outside the scope of the current manuscript. However, the engineering design and *in vitro* validation described herein represents the first known losartan-controlled release system, which may have broad implications across multiple healthcare fields as losartan is a well-established oral drug for lowering blood pressure.

Taken together the results of this study suggest that the positively charged K-PA nanofibers bound with moderate (5 mg/mL) losartan can have a pro-regenerative phenotype in OA-activated chondrocytes by inhibiting *tgfβ1* expression. This therapeutic strategy for articular cartilage repair using losartan sustained release PA nanofibers may provide a safer and more efficient treatment compared to oral administration or intra-articular injection of losartan. A challenge with adapting the route of administration and including any drug delivery platform, including the PA nanofibers, is that clinical use of losartan in this formulation will require Food and Drug Administration review as an Investigational New Drug as a combination product. However, given the significant burden of disease of OA and the established role for losartan in addressing cardiovascular disease, such efforts could be justified.

## Conclusion

The results of this study showed that positively charged K-PA nanofibers with moderate (5 mg/mL) sustained release of losartan have anti-inflammatory and anti-degenerative effects on chondrocytes. Their regenerative effect is asserted mechanistically by promoting cell proliferation and chondrocyte GAG production, while suppressing hypertrophic differentiation. This therapeutic strategy for articular cartilage repair using losartan sustained release PA nanofibers may provide a safer and more efficient treatment for local administration of the drug.

## Data Availability

The raw data supporting the conclusion of this article will be made available by the authors, without undue reservation.
